# Medical Officers and Workhouse Nursing

**Published:** 1899-07-08

**Authors:** 


### MEDICAL OFFICERS AND WORKHOUSE NURSING.

There is a little place in Ireland called Limavady, the Poor Law Guardians of which have unintentionally done a good turn to the poor in general, for by their obstinacy they have led the Local Government Board to make plain the way in which trained nursing can be secured for the sick in workhouses. It appears that the Guardians advertised for a trained nurse, offering a salary of £35 per annum, but obtained no response; so they let the matter drop, and went on as they were. The Local Government Board then advised them to try again, offering a higher salary, but this they declined to do. On this the Board wrote to the medical officer of the workhouse, saying that they would hold him accountable for the nursing arrangements of the workhouse, and that as, in their opinion, at least one trained or highly-qualified nurse was necessary, he should at once send a requisition to the workhouse master for such a nurse; and at the same time they wrote to the master saying that they would hold him responsible for obtaining the services of such a trained or qualified nurse on receipt of a requisition from the medical officer. Of course, the Board of Guardians are very angry; but we fancy that they will find the Local Government Board too strong for them.

